# High prevalence of intrathecal IgA synthesis in multiple sclerosis patients

**DOI:** 10.1038/s41598-022-08099-y

**Published:** 2022-03-11

**Authors:** Úrsula Muñoz, Cristina Sebal, Esther Escudero, Maria Isabel García Sánchez, Elena Urcelay, Asier Jayo, Rafael Arroyo, Maria A. García-Martínez, Roberto Álvarez-Lafuente, María C. Sádaba

**Affiliations:** 1 Facultad de Medicina, Instituto de Medicina Molecular Aplicada (IMMA), Universidad San Pablo-CEU, CEU Universities, Crta Boadilla del Monte Km 5,3, Madrid, Spain; 2 UGC Neurología (Biobanco Hospitalario), Hospital Universitario Virgen Macarena, Red Española de Esclerosis Múltiple (REEM), Madrid, Spain; 3 Instituto de Investigación Sanitaria San Carlos (IdISSC)/Hospital Clínico San Carlos, Madrid, Spain; 4 Departamento de Neurología, Hospital Universitario Quironsalud, Madrid, Spain; 5 Grupo de Investigación de Factores Ambientales en Enfermedades Degenerativas, Instituto de Investigación Sanitaria San Carlos (IdISSC)/Hospital Clínico San Carlos, Madrid, Spain

**Keywords:** Biochemistry, Biological techniques, Immunology, Neuroscience, Neurology

## Abstract

The detection of intrathecal IgA synthesis (IAS) in multiple sclerosis (MS) could be underestimated. To assess it, we develop a highly sensitive assay based on isoelectric focusing (IEF). 151 MS patients and 53 controls with different neurological diseases were recruited. IgA concentration was analyzed using a newly developed in house ELISA. IgA oligoclonal bands to detect IAS were determined by IEF. Most individuals showed an IgA concentration within normal range in serum samples (90.69%) but 31.37% of individuals had a IgA concentration below the normal range in the cerebrospinal fluid (CSF). No significant differences were observed between MS and control groups, neither in CSF nor in serum. The new IEF was more sensitive than those previously described (0.01 mg/dl of IgA), and clearly identified patients with and without IAS, that was not related with IgA concentration. Using IEF, MS patients showed higher percentage of IAS-IEF (43.00%) than the control group (16.98) (p = 0.001). The incidence was especially higher in patients with clinically isolated syndrome (66.00%). The new IFE demonstrated a higher percentage of IAS in MS patients than assumed in the past. The presence of IAS-IEF in MS is higher than in other neurological diseases.

## Introduction

The detection of intrathecal (cerebrospinal fluid, CSF) synthesis of IgG^1–3^ and IgM^4,5^ are hallmarks for the diagnosis and prognosis of multiple sclerosis (MS) patients. Intrathecal synthesis of these immunoglobulins is detected using quantitative and qualitative methods. The latter, based on the identification of oligoclonal bands in CSF vs. paired serum, is more sensitive than the former^6^.

In contrast, the detection of IgA in MS patients is not a diagnostic biomarker in MS. Although quantitative analysis of the CSF demonstrated that 18% of patients present intrathecal IgA synthesis (IAS)^7^, and qualitative analysis of these samples showed similar results^8^, there are not many reports about the role of IgA in MS patients. Moreover, quantitative analysis of IgA is not routinely performed in Spanish hospitals.

IgA is a main response to viruses^9–13^, and they have been related to MS in the last years^14–16^. Moreover, IgA-positive plasma cells present in the central nervous system (CNS) tissues of MS patients were claimed to be the main source of IgA deposits along axons detected in the lesions, which in turn, were related with axonal damage^17^. Furthermore, IgA-positive lymphocytes have also been detected in the CSF from MS patients^18^.

However, both the very low IgA concentration in CSF^19^, and being a highly glycosylated dimeric immunoglobulin, hamper the establishment of quantitative and qualitative analyses. For this reason, we aimed to develop an ultrasensitive and specific assay for the detection of oligoclonal IgA bands (OGIgAB) in CSF and serum samples in order to study the intrathecal IgA synthesis in MS patients.

## Materials and methods

### Samples

Samples were obtained from, Hospital Clínico San Carlos, Hospital Universitario Quirónsalud (Madrid) and Biobanco del Hospital Universitario Virgen Macarena. Biobanco del Sistema Sanitario Público de Andalucía (B.B.S.S.P.A.).

We analyzed serum and CSF paired samples from 151 MS patients and 53 controls, which included 30 individuals with non-demyelinating neurological diseases (NDND) and 23 patients with non-MS demyelinating neurological diseases (NonMSDND). NDND group comprised individuals suffering headache (5), amyotrophic lateral sclerosis (2), epilepsy (3), schizophrenia (1), cranial hypertension (2), Non-Hodgkin lymphoma (1), lymphocytic meningitis (1), meningococcal meningitis (1), autoimmune myelopathy (1), secondary myelopathy to tumor (1), cerebellar syndrome (1), capillary telangiectasia (1), spastic paraparesis/HTLV-1–associated (1), stroke (1), anti-NMDA receptor encephalitis (1), Parkinson (1), neurosarcoidosis (2), hyper-IgE syndrome related myelopathy (1), myelopathy (1), small cell lung carcinoma related neuritis (1) and Sjögren's syndrome related neuritis (1). NonMSDND group included patients with myelitis (10), optic neuritis (8) and polyneuropathy (5).

All specimens were collected for clinical purposes, and the remaining material was stored at – 80 °C until experiments were performed. The demographic and clinical data of all individuals are summarized in Table 1.

**Table 1:** Demographic and clinical data from patients with MS, NonMSDND and NDND.

Disease	Females	Age (mean (minimum–maximum)	Disease duration	EDSS score	Treatment
**MS (151)**	64.9% (98)	40.0 (13–80)	9.9 (0–41)	3.5 (0–8.5)	17.9% (27)
CIS (56)	67.9% (38)	34.89 (13–60)	2.9 (0–13)	1.7 (0–6.5)	0% (56)
RRMS (50)	60.0% (30)	38.1 (18–56)	8.9 (0–28)	1.8 (0–4.5)	30% (15)
SPMS (24)	70.8% (17)	45.8 (29–67)	27.1 (15–41)	6.1 (3.5–8.5)	33.3% (8)
PPMS (21)	61.9% (13)	51.7 (33–80)	11.6 (1–35)	4.9 (2.5–7.5)	19% (4)
**CONTROLS (53)**					
NonMSDND (23)	43.48% (10)	39.00 (14–72)			
Myelitis (10)					
Optic neuritis (8)					
Polyneuropathy (5)					
NDND (30)	70.00% (21)	43.10 (2–78)			
Headache (5),					
Amyotrophic lateral sclerosis (2)					
Epilepsy (3)					
Schizophrenia (1)					
Cranial hypertension (2)					
Non-Hodgkin lymphoma (1)					
Lymphocytic meningitis (1)					
Meningococcal meningitis (1)					
Autoimmune myelopathy (1)					
Secondary myelopathy to tumor (1)					
Cerebellar syndrome (1)					
Capillary telangiectasia (1)					
Spastic paraparesis/HTLV-1–associated (1)					
Stroke (1)					
Anti-NMDA receptor encephalitis (1)					
Parkinson (1)					
Neurosarcoidosis (2)					
Hyper-IgE syndrome related myelopathy (1)					
Myelopathy (1),					
Small cell lung carcinoma related neuritis (1)					
Sjögren's syndrome related neuritis (1)					

*MS* multiple sclerosis, *CIS* clinically isolated syndrome, *RRMS* relapsing–remitting multiple sclerosis, *SPMS* secondary progressive multiple sclerosis, *PPMS* primary progressive multiple sclerosis, *NonMSDND* non-MS demyelinating neurological diseases, *NDND* non-demyelinating neurological diseases, *Females and treatment* percentage and total number; Age, disease duration and EDSS score: mean (minimum and maximum. Patients were treated with Natalizumab (8), fingolimod (3), interferon-β (8), Copaxone (1), dimethyl fumarate (1) and 8 cases were included in a clinical trial.

All methods were carried out in accordance with relevant guidelines and regulations.

All the protocols were approved by Committee of Bioethics of Hospital Clínico Universitario, Committee of Bioethics of Hospital Universitario Quirónsalud and Committee of Investigación Biomédica de la Junta de Andalucía.

The informed consent was obtained from all the patients and/or their legal guardian(s) (As participants less than 16 years are also included).

The diagnosis of MS patients was established according to 2010 McDonald criteria (McDonald 2010). MS patients were classified as CIS (clinically isolated syndrome), RRMS (relapsing remitting MS), and PPMS (primary-progressive MS) when the sample was obtained. All CIS patients analyzed develop MS.

### Analysis of IgA concentration in CSF and serum samples by ELISA assay

We developed a quantitative assay to measure IgA concentrations in serum and CSF samples. Briefly, 400 ng of anti-human IgA (Jackson ImmunoResearch) diluted in 100 µL of phosphate buffered saline solution (PBS, Sigma) were added to every well of the microtiter plate (Greiner). After overnight incubation at 4 °C, the plates were washed and blocked with 200 µL of bovine albumin (Sigma) diluted at 3% in PBS. To perform the standard curve, we added to the first row twofold serial dilutions (from 40 to 0.08 ng/mL) of IgA (NProtein Standard SL, Siemens). Then, twofold serial diluted samples (from 1:200 to 1:1600) were added to the wells. After overnight incubation, 100 µL of biotinylated anti-human IgA (Jackson ImmunoResearch) diluted (1:10,000) in blocking solution was added to the plates and incubated for 2 h. Next, we added 100 µL of streptavidin-HRP (horseradish peroxidase, Jackson ImmunoResearch) diluted (1:1000) in blocking solution. After 30 min incubation, color was developed using TMBone (Thermofisher) substrate. Finally, the reaction was stopped using 100 µL of H_2_SO_4_ (Sigma Aldrich) diluted at 5% in distilled water, and plates were read in the Varioscan instrument (Thermofisher). Graphpad Prism v8 software was used to obtain all he standard curves and to calculate the IgA concentration in all samples analyzed.

In order to validate the ELISA, we analyzed the results of 15 ELISAs. To assess the linearity, the limit of blank [LoB, mean_blank + 1.645*SD_blank (standard deviation)] and reproducibility of the ELISA, we analyzed the absorbances of the standard curves and the blank rows. The absorbances obtained at IgA concentrations as low as 0.3125 ng/mL were analyzed to obtain the limit of detection (LoB + 1645*SD_low concentration samples). To study the coefficient of variation of the ELISA we analyzed the IgA concentration obtained using a control (N/T Protein Control SL, Siemens) at twofold dilutions (from 20 to 0.08 ng/mL) (Supplementary Data 1).

The data obtained from the analysis of the samples were compared with the normal IgA concentration in serum (70–400 mg/dL)^20^ and LCR (0.06–0.4 mg/dL)^19^.

### Reibergram

Serum and CSF albumin were analyzed in a Beckman-Coulter AU5400 instrument (Beckman Coulter), using the Olympus Albumin Reagent Test (Beckman Coulter) and the Urine CSF Album KIT (Beckman Coulter) when serum and CSF samples were analyzed respectively.

Software by Laboratory Enders & Partners (www.labor-enders.de/19.html?&L=1) was used to calculate the Reibergram.

### Isoelectric focusing (IEF) and immunodetection assay for the detection of oligoclonal IgA bands in CSF and serum samples

The protocol used was similar to that previously described by our group^1^, but we introduced the modifications described below.

We incubated 40 µL of CSF and serum samples with 5 µL of 0.5 M dithiothreitol and 5 µL of 1 M Tris/HCl pH 9.7, for 45 min.

For protein separation, we used an agarose gel consisting of 0.3 g of agarose (GE Healthcare); 3.6 g of sorbitol (Sigma-Aldrich); 1.25 mL of each Pharmalyte, pH 4–6.5 and pH 3–10 (GE Healthcare); 2.5 mL of glycerol, and 22.5 mL of water.

To detect IgA, we used a biotinylated anti-human IgA antibody (Jakson Immunoresearch) diluted (1:20.000) in the blocking solution. After incubating the membrane overnight at 4 °C, the labeling was developed using streptavidin-HRP (Jakson Immunoresearch) diluted (1:1.000) in blocking solution.

To study the sensitivity of this new assay (IEF and immunodetection), we analyzed paired CSF and serum samples from 3 MS patients showing OGIgAB. We applied 5 µL of twofold serial diluted samples (from 2 to 0 ng of IgA) on the gel in triplicate.

Representative full blots including the patterns of OGIgAB represented in pictures 1 and 2 are supplied in Supplementary Data 1.

To assay the limit of blank and the limit of detection, diluted CSF and serum samples with an extremely low quantity of IgA (0 ng and 0.25 ng respectively) were analyzed.

### Image processing

ImageJ1 (ImageJ, U. S. National Institutes of Health, Bethesda, Maryland, USA) was used to manage the images, and to quantify the staining signal to validate the OGIgAB pattern. Three measurements were obtained from every sample.

### Statistics

IBM SPSS v24.0 software was used to analyze the data obtained. To analyze the linearity of both assays, ELISA and IEF and immunodetection, spearman test was used to analyze two different correlations, between IgA concentration and absorbance, and between IgA concentration and relative optic density (O.D). In addition, linear regression was performed to study the distribution of the residuals and the collinearity of the data. Linearity of the assay was accepted when variance inflation factor (VIF) < 20. The coefficient of variation represents the percentage of the standard deviation divided by the mean. To analyze the IgA concentration in CSF and serum samples Mann–Whitney *U* test was used to compare MS and control patients, and Kruskal–Wallis for comparisons among different MS types. To analyze the fraction of individuals harboring intrathecal IgA synthesis (IAS), we used the Fisher test for comparisons between MS and control group and the Pearson χ^2^ test for comparisons among different MS types. Statistically significant differences were accounted when p < 0.05.

To calculate the specificity of the detection of IAS in MS we used the equation: TN/[TN + FP]) × 100 (TN: True Negative. FP: False Positive).

## Results

### Analysis of the IgA concentration in CSF and serum samples: reibergram

Once validated the ELISA developed in our lab (Supplementary S1, Supplementary Fig. 2), we analyzed the samples from MS patients and control group. The IgA concentration in CSF and serum samples is summarized in Table 2.

**Table 2:** Analysis of the IgA concentration in CSF and serum samples from MS patients and control groups.

	NonMSDND	NDND	MS	CIS	RR	SP	PP
	(n = 23)	(n = 30)	(n = 151)	(n = 56)	(n = 50)	(n = 24)	(n = 21)
**Serum IgA (mg/dL)**							
Mean ± SEM	272.46 ± 42.39	224.84 ± 28.54	144.95 ± 8.55	162.74 ± 16.34	137,72 ± 13.75	108.99 ± 12.13	155.81 ± 23.70
Minimum	30.23	6.25	9.43	9.43	19.01	31.70	19.00
Maximum	959.16	458.87	764.47	764.47	646.10	256.00	427.00
**CSF IgA (mg/dL)**							
Mean ± SEM	0.232 ± 0.035	0.195 ± 0.026	0.71 ± 0.60	0.11 ± 0.015	0.101 ± 0.014	0.070 ± 0.015	4.48 ± 4.28
Minimum	0.084	0.012	0.002	0.002	0.006	0.010	0,01
Maximum	0.632	0.504	90.09	0.51	0.620	0.295	90.09
**Serum albumin (g/L)**							
Mean ± SEM	4.03 ± 0.10	4.18 ± 0.09	4.42 ± 0.04	4.56 ± 0.06	4.47 ± 0.06	4.21 ± 0.09	4.15 ± 0.09
**CSF albumin (mg/L)**							
Mean ± SEM	21.16 ± 2.46	21.31 ± 4.19	20.68 ± 0.66	19.20 ± 0.92	20.17 ± 1.03	24.79 ± 2.29	21.14 ± 1.62
**Positive IAS calculated by Reibergram**							
	14.29%	14.29%	16.78%	16.07%	12.50%	16.67%	23.81%

*NonMSDND* Non-MS demyelinating neurological diseases, *NDND* non-demyelinating neurological diseases, *MS* multiple sclerosis patients, *CIS* clinically isolated syndrome, *RRMS* relapsing–remitting multiple sclerosis, *SPMS* secondary progressive multiple sclerosis, *PPMS* primary progressive multiple sclerosis, *SEM* standard error of the mean, *IAS* intrathecal IgA synthesis.

Most of the studied individuals (185/204) showed a serum IgA concentration within the normal range (n.r). We observed a serum IgA concentration over the n.r in 3/151 MS patients, who had an IgA level in the CSF within the n.r. 9/53 patients from the control group showed a serum IgA concentration higher than the n.r, but only 4 had an IgA concentration in CSF above the n.r. Low serum IgA concentration (< 20 mg/mL) was detected in 2/53 individuals from the control group and in 5/151 MS patients, all of them showed an IgA concentration in CSF lower than the n.r.

However, only 62.75% of the individuals (128/204) showed an IgA concentration in CSF within the n.r previously described in patients without neurological diseases. 5 MS patients showed an IgA concentration in CSF over the n.r, but they had serum IgA concentration within the n.r. We observed IgA concentrations in CSF below the n.r in 6/53 individuals from the control group, and in 58/151 MS patients. From those, only 2 individuals from the control group and 5 MS patients showed a serum IgA concentration below the n.r.

No statistically significant differences in IgA concentration were detected neither in CSF nor in serum when MS and control group were compared. Neither did we detect differences among MS patients in different stages of the diseases.

Then, we analyzed the presence of IAS using the Reibergram (Table 2). We did not detect statistical significances between MS patients and control group in the percentage of IAS when the Reibergram was used. Neither did we detect differences among the different MS types.

### Detection of OGIgAB by a new isoelectric focusing and immunodetection assay

**Figure 1:**
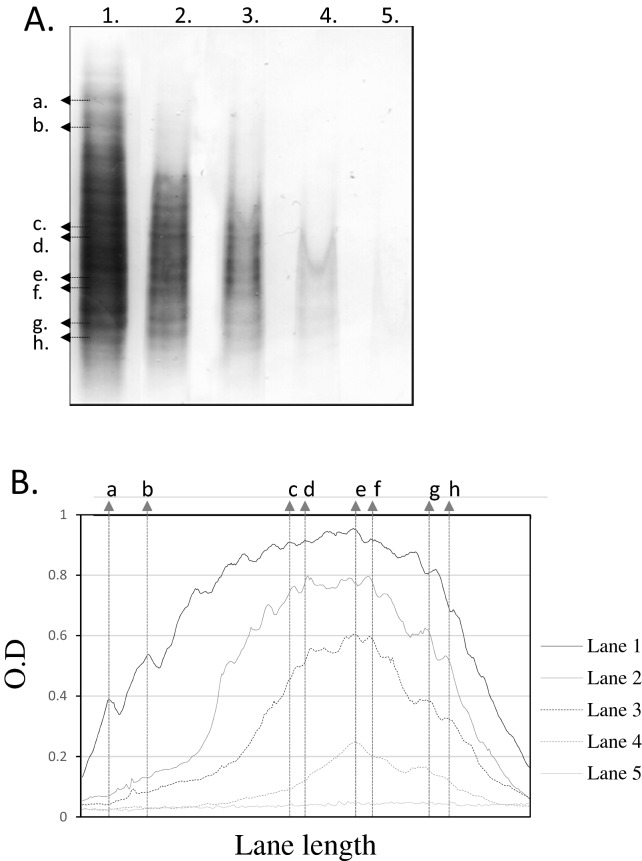
IEF and immunodetection of OGIgAB in a diluted serum sample from a MS patient. The quantity of IgA in lines 1–5 was: 4, 2, 1, 0.5 and 0.25 ng, respectively. (**A**) OGIgAB were observed in lines 1–4. but not in line 5. (**B**) The densitometric analysis of the band pattern in lines 1–4 showed a similar and reproducible OGIgAB pattern in all the lanes quantified. Arrows show specific bands (**A**) and its corresponding densitometric signals in the linescan analysis (**B**). Note that a single band corresponds to a density peak in the linescan analysis in most of the lanes. O.D. is represented as relative O.D., the value obtained divided by maximum.

Once demonstrated the linearity, accuracy, and reproducibility of the new assay (Supplementary Data 3), we studied the sensitivity of the method to detect OGIgAB. The visual analysis of the western blots demonstrated that it was possible to detect OGIgAB when the amount of IgA applied on the gel was 0.5 ng (Fig. 1A). Polyclonal IgA but no OGIgAB were detected when the amount of IgA applied on the gel was 0.25 ng. IgA was not detected when the amount of IgA applied on the gel was 0.125 ng. To validate these results, we quantified the optic density per area (pixels) of the whole range of dilutions (Lanes 1–5). The densitometric analysis also showed that the OGIgAB pattern (peaks a–h) is reproducible and detectable from Lane 1 to 3. No peaks were observed in lanes 4–5 (Fig. 1B). In addition, the sensitivity of the IEF and immunodetection assay to detect OGIgAB is 5 times higher than the limit of blank (0.095 ng of IgA) and 2 times higher than the limit of detection (0.236 ng of IgA).

Then, we analyzed if the assay was sensitive enough to detect the presence of OGIgAB in all CSF samples. This new assay can detect OGIgAB when the IgA concentration in CSF or serum samples is equal or higher than 0.01 mg/dL. Considering the IgA concentration in our cohort (Table 2), and supposing a Gaussian distribution, the 99.9% of the population would have an IgA concentration higher than 0.05 mg/dL [mean ± 3.291 × SEM (standard error of the mean)].

Once demonstrated that the new IEF and immunodetection assay could detect up to five times more than required, we analyzed the presence of OGIgAB in samples from MS patients and control group.

**Figure 2:**
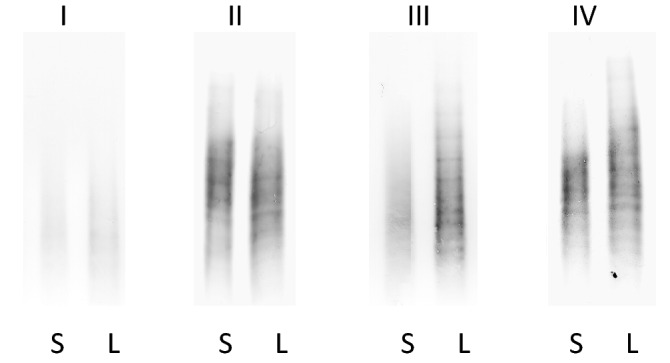
Detection of OGIgAB in paired CSF and serum samples from 4 MS patients. Representative blottings of individuals showing Pattern 1 (I), Pattern 2 (II), Pattern 3 (III) and Pattern 4 (IV). S: serum. L: cerebrospinal fluid.

Then, we analyzed paired CSF and serum samples from both, control group and MS patients, and we classified the four patterns observed as described previously by Freeman et al.^21^ (Fig. 2):

- Pattern I: Polyclonal IgA in serum and in CSF.
- Pattern II: OGIgAB in CSF, but not in serum
- Pattern III: OGIgAB in serum, and two or more additional OGIgAB in CSF.
- Pattern IV: OGIgAB in serum and the same OGIgAB in CSF.

The incidence of these patterns is described in Table 3. Using IEF, we grouped the individuals in two categories: patients without (Pattern I and Pattern IV) and with (Pattern II and Pattern III) intrathecal IgA synthesis (IAS-IEF) (Table 3). We detected a higher percentage of IAS-IEF in of MS patients (43.00%) than in control group (16.98%; p = 0.001).

**Table 3:** Detection of OGIgAB in paired CSF and serum samples from MS and control patients.

	Pattern I	Pattern II	Pattern III	Pattern II
NonMSDND (n = 32)	17.39% (4)	8.70% (2)	0% (0)	73.91% (17)
NDND (n = 21)	13.33% (4)	10.00% (3)	13.33% (4)	66.33% (19)
MS (n = 151)	21.90% (33)	12.60% (19)	30.50% (46)	35.10% (53)
CIS (n = 56)	16.10% (9)	19.60% (11)	46.40% (26)	17.90% (10)
RRMS (n = 50)	32.00% (16)	6.00% (3)	28.00% (14)	34.00% (17)
SPMS (n = 24)	20.80% (5)	16.70% (24)	20.80% (4)	41.7% (10)
PPMS (n = 21)	14.30% (3)	4.80% (1)	4.80% (1)	76.20% (16)

*NonMSDND* Non-MS demyelinating neurological diseases, *NDND* non-demyelinating neurological diseases, *MS* multiple sclerosis, *CIS* clinically isolated syndrome, *RRMS* relapsing–remitting multiple sclerosis.

Then, we studied the specificity of the detection of IAS-IEF in MS, which reached 83.02%.

To study in detail the sensibility and specificity of the assay, we analyzed the presence of IAS in individuals with low and high concentration of IgA in CSF.

OGIgAB were detected only in 2/12 CSF samples with high IgA concentrations. However, we could detect OGIgAB in 20/64 CSF samples showing low IgA concentration, and 20 of these individuals had IAS-IEF. The individuals from the control group showing IAS (9/53) were patients with intracranial hypertension, Non-Hodgkin lymphoma, epilepsy, myelitis, spastic paraparesis/HTLV-1-associated, polyneuropathy, small cell lung carcinoma related neuritis, Sjögren's syndrome related neuritis, hyper-IgE syndrome related myelopathy and amyotrophic lateral sclerosis.

### Presence of IAS in the different phases of the disease measured by the detection of OGIgAB

We also aimed to analyze the relation IAS-IEF and the disease course. CIS patients showed a significant higher percentage of IAS (66.07%) than RRMS (34.00%; p = 0.002), SPMS (37.50%; p = 0.026); PPMS (9.50%; p = 0.001) and control subgroups NonMSDND (8.70%; p = 0.001) and NDND (23.33%; p = 0.001). We also detected a higher percentage of IAS-IEF in RRMS than in PPMS (p = 0.041) or NonMSDND (p = 0.024). The percentage of patients with IAS-IEF was higher in SPSS when compared with PPMS (p = 0.040) or NonMSDND (p = 0.036) groups.

**Figure 3:**
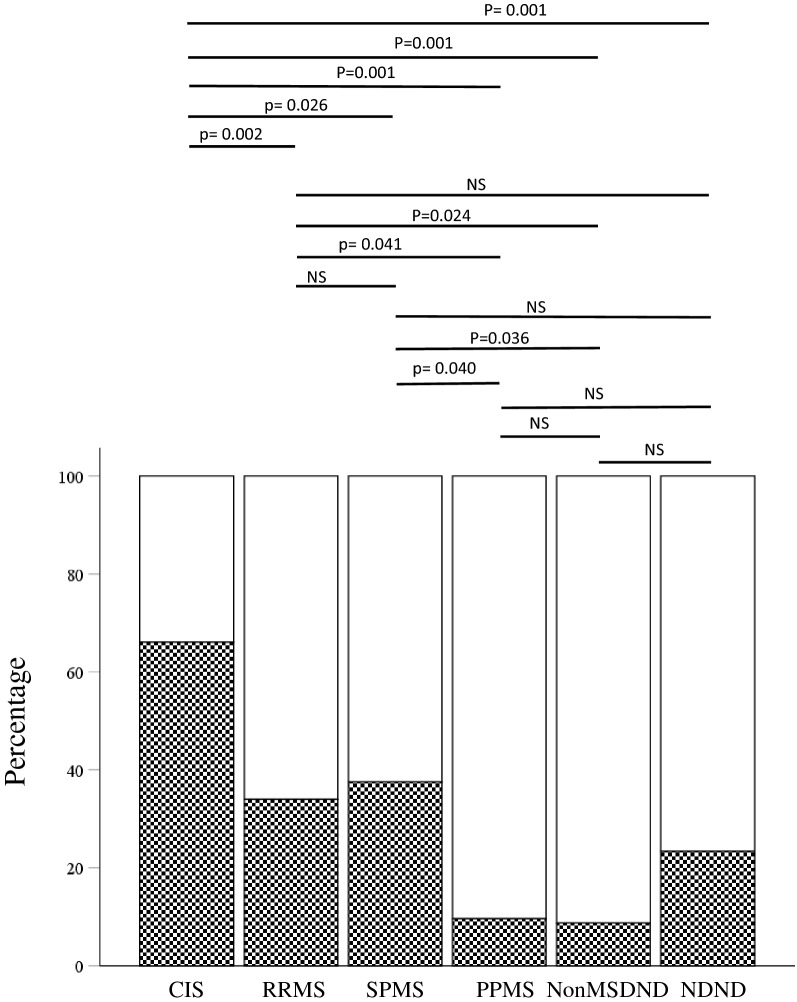
Percentage of intrathecal IgA synthesis in MS groups using isoelectric focusing and immunodetection. White area represents the percentage of individuals of each group without IAS. Squared areas represent the percentage of individuals with IAS. *CIS* clinically isolated syndrome, *RRMS* relapsing–remitting patients.

No statistical significance was detected between PPMS and NonMSDND. Neither did we detect differences when RRMS, SPMS, PP and NDND were compared (Fig. 3).

## Discussion

There are not many reports about the contribution of intrathecal IgA in MS patients, because it is supposed that the incidence is low^7^. It was reported that qualitative methods are the most sensitive methods to detect intrathecal immunoglobulin synthesis^7^, but the detection of IgA by these methods is difficult^19^. For these reasons, we aimed to develop a new method based on IEF and immunodetection to detect OGIgAB in CSF and serum samples to analyze the presence of IAS in MS patients.

First, we developed a highly sensitive quantitative ELISA to analyze the IgA concentration in CSF and serum samples. Once demonstrated the linearity, accuracy, sensitivity, and reproducibility of the ELISA, we analyzed the concentration of this immunoglobulin in samples from patients with MS and different neurological diseases.

In our cohort, IgA concentration in serum samples was found within normal ranges in most cases. The analysis of CSF showed an IgA concentration within the previously described normal range^19^ in most cases, but a considerable number of the individuals (30.33%) had a low IgA concentration, which made difficult to detect IAS by classical methods. We did not detect significant differences in the IgA concentration between patients with MS or different neurological diseases, neither in CSF nor in serum samples. Neither did we detect statistical differences when the different MS types were compared.

Then we analyzed the presence of IAS by a quantitative method, the Reibergram, and we observed that the percentage of MS patients showing IAS using this method was similar to described previously^7^.

Then, we developed a new IEF to detect OGIgAB, considering the concerns of the separation of IgA. It was described that the proportion of dimeric IgA increases from 5%, up to 53.9% when control and MS patients were compared^22^. Therefore, we reduced the samples with DTT to obtain monomeric IgA. Second, we employed agarose instead of acrylamide gels to avoid the IgA precipitation^19^. Finally, we used alkaline phosphatase to develop the staining, this enzyme improves tenfold the sensitivity of the assay compared with peroxidase^1^.

Once established the best conditions, we demonstrated the linearity, accuracy, and reproducibility of the IEF and immunodetection. This sensitivity is higher than described previously^8^, and taking into account the normal range of IgA in the CSF, is high enough to detect OGIgAB in most of the CSF samples. For this reason, we observed a higher prevalence than previously described. The new assay showed four different patterns. Pattern I (polyclonal IgA in CSF and serum) indicates that all the B-lymphocytes release a limited amount of antibody because they are not stimulated, this is the normal situation. Pattern II (presence of OGIgAB in CSF but not in serum) demonstrates the presence of B-lymphocytes releasing this immunoglobulin in the CNS. Pattern III (two or more OGIgAB in CSF compared with serum) discloses the presence of activated B-lymphocytes in the CNS, but also indicates that some are also activated in the peripheral system. Pattern IV (the same OGIgAB in CSF and serum) involves activated peripheral B-lymphocytes producing this immunoglobulin, which finally diffuses through the blood CSF barrier into the CNS. According to these patterns, we could identify IAS in a higher percentage than described before for MS patients^22^ This new assay is more sensitive than Reibergram to detect IAS, corroborating that qualitative techniques are more sensitive than quantitative methods^6,23^. The presence of OGIgAB is not related with IgA concentration. We detected OGIgAB in individuals with an IgA concentration in CSF below the normal range. On the contrary, most of the patients with an IgA concentration in CSF above the normal range did not show OGIgAB.

Using this new assay, the incidence of IAS-IEF was higher in MS patients, compared with other demyelinating diseases or non-demyelinating neurological diseases. Most of the MS patients with IAS-IEF showed Pattern III, in contrast to the situation observed when IgG or IgM oligoclonal bands are analyzed^1,24^. Therefore, the presence of additional oligoclonal bands in CSF (Pattern III) is the most frequent pattern. Nevertheless, a considerable percentage of MS patients showed Pattern IV. This was not expected, because this pattern is not usually observed when IgG or IgM oligoclonal bands are analyzed in MS patients^1,24^. The incidence of Pattern IV was particularly high in NonMSDND and NDND. These data, and those obtained from the analysis of IgG and IgM in the CSF indicate a characteristic humoral immune response in MS, compared with other demyelinating and non-demyelinating diseases.

In addition, these data suggest that IgA, as the other immunoglobulins, could have a dual role in MS, protecting against the disease or promoting the cell damage.

Regarding the protective role of IgA in MS, it was recently described that recirculating intestinal IgA positive cells prevent the development of experimental autoimmune encephalomyelitis, the animal model of MS, because they release IL-10^25^. We have described above the high incidence of OGIgAB in serum samples (Patter III and IV) from MS patients, demonstrating the presence of peripheral activated B lymphocytes in a high percentage of these individuals, more than 60% in both, CIS and RRMS patients.

Moreover, IgA is involved in the first line of defense against pathogens, and increases of the viral DNA prevalence and of the IgG viral titers in MS patients have been previously found. For example, our group and others have previously detected higher amounts of viral DNA of human herpesvirus 6, JC virus and Epstein Barr virus (EBV) in the CSF of MS patients than in controls^26–28^, and infections have been associated with a an increased risk of exacerbations (rate ratio 2.1)^29^. Furthermore, it has been recently published that the risk of MS increased 32-fold after infection with EBV.

Nevertheless, the activation of the immune system could trigger cross-reactions against self-antigens, one of the classical hypothesis in autoimmunity^30^. For example, it has been recently published a high-affinity molecular mimicry between the EBV transcription factor EBNA1 and the CNS protein GlialCAM, showing a possible mechanistic link for the association between MS and EBV^14^. Regarding IgA, deposits of this immunoglobulin observed in demyelinated areas of MS patients were related with axonal damage^17^.

In summary, here we present a highly sensitive technique to detect OGIgAB in CSF and serum samples. Our findings draw attention to the high prevalence of IAS in MS patients, especially in CIS group. Nevertheless, the study has limitations, controls do not include a high number of infectious diseases, it is a new technique without external confirmation, and it is a retrospective study, so remains unknown the presence of OGIgAB in CSF along the evolution of the disease. Further studies would be of great interest to clarify the role of this immunoglobulin in the physiopathology of MS.

## Supplementary Information

Supplementary Information 1.

Supplementary Information 2.

Supplementary Information 3.
