# Supplementary material for: High prevalence of intrathecal IgA synthesis in multiple sclerosis patients
Source: Sci Rep. 2022 Mar 11;12:4247. doi: 10.1038/s41598-022-08099-y (PMC8917141; doi:10.1038/s41598-022-08099-y)
Supplement: Supplementary file 1 — Supplementary Information 1. [file 41598_2022_8099_MOESM1_ESM.doc]

TITLE PAGE.

**Title: High prevalence of intrathecal IgA synthesis in multiple sclerosis patients**

Úrsula Muñoz PhD^1^, Cristina Sebal Phd^1^, Esther Escudero PhD^1^, Maria Isabel García Sánchez PhD^2^, Elena Urcelay PhD^3^, Asier Jayo PhD^1^, Rafael Arroyo MD PhD^4^, Maria A García-Martínez LA^5^, Roberto Álvarez-Lafuente PhD^5^, María C Sádaba, PhD^1^.

^1^ Facultad de Medicina, Instituto de Medicina Molecular Aplicada (IMMA), Universidad San Pablo-CEU, CEU Universities. Crta Boadilla del Monte Km 5,3. Madrid. Spain.

^2^ UGC Neurología (Biobanco Hospitalario), Hospital Universitario Virgen Macarena, Red Española de Esclerosis Múltiple (REEM), Madrid, Spain.

^3^ Instituto de Investigación Sanitaria San Carlos (IdISSC) / Hospital Clínico San Carlos, Madrid. Spain.

^4^Departamento de Neurología. Hospital Universitario Quironsalud Madrid. Spain

^5^Grupo de Investigación de Factores ambientales en enfermedades degenerativas. Instituto de Investigación Sanitaria San Carlos (IdISSC) / Hospital Clínico San Carlos. Madrid. Spain.

**Correspondence to:**

Maria Cruz Sadaba Argaiz

Facultad de Medicina, Instituto de Medicina Molecular Aplicada (INMA), Universidad San Pablo-CEU, CEU Universities. Crta Boadilla del Monte Km 5,3. Madrid. Spain.

Email: [mariacruz.sadabaargaiz@ceu.es](mailto:mariacruz.sadabaargaiz@ceu.es)

Telephone: (+34) 914566300

Roberto Álvarez Lafuente

Grupo de Investigación de Factores ambientales en enfermedades degenerativas. Instituto de Investigación Sanitaria San Carlos (IdISSC) / Hospital Clínico San Carlos. Madrid. Spain.

Email: [ralvarezlafuente@yahoo.es](mailto:ralvarezlafuente@yahoo.es)

Telephone: (+34) 913303726

Keywords: multiple sclerosis, oligoclonal bands, isoelectrofocusing, intrathecal IgA synthesis.

DISCLOSURE OF CONFLICTS OF INTEREST

Authors have nothing to disclosure.

LEGEND:

Supplementary fig.2. Representative images of full immunoblots. A) Representative image of Pattern I and Pattern II. B) Representative image of Pattern III. C) Representative image of pattern IV.


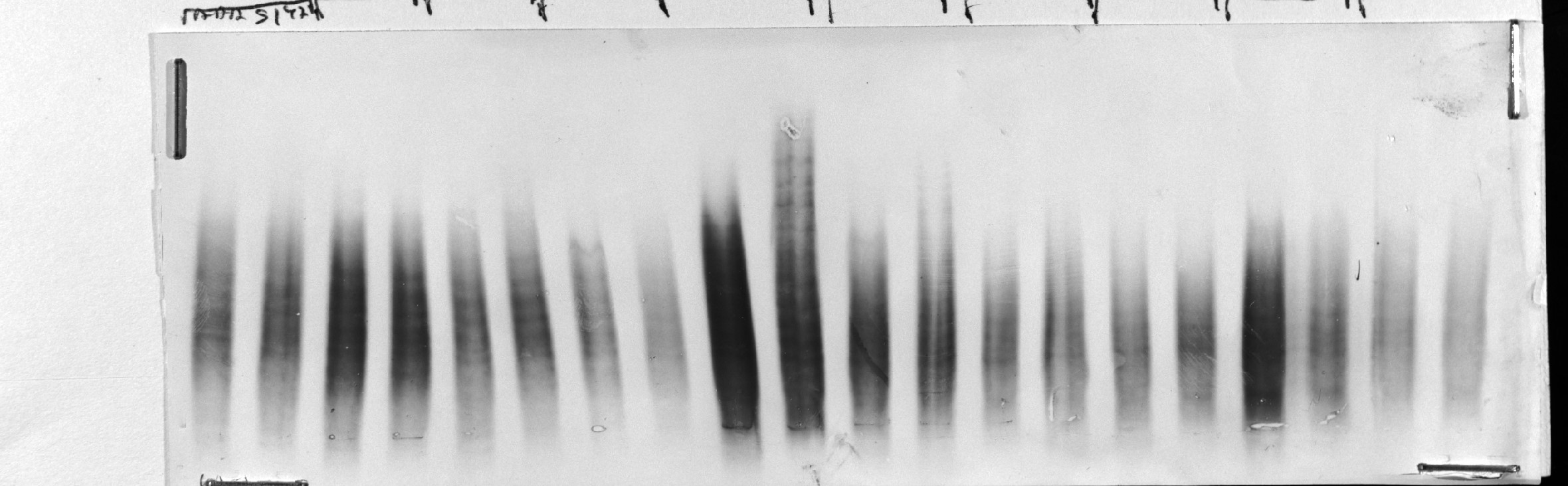


**I**

**II**

A

**00**


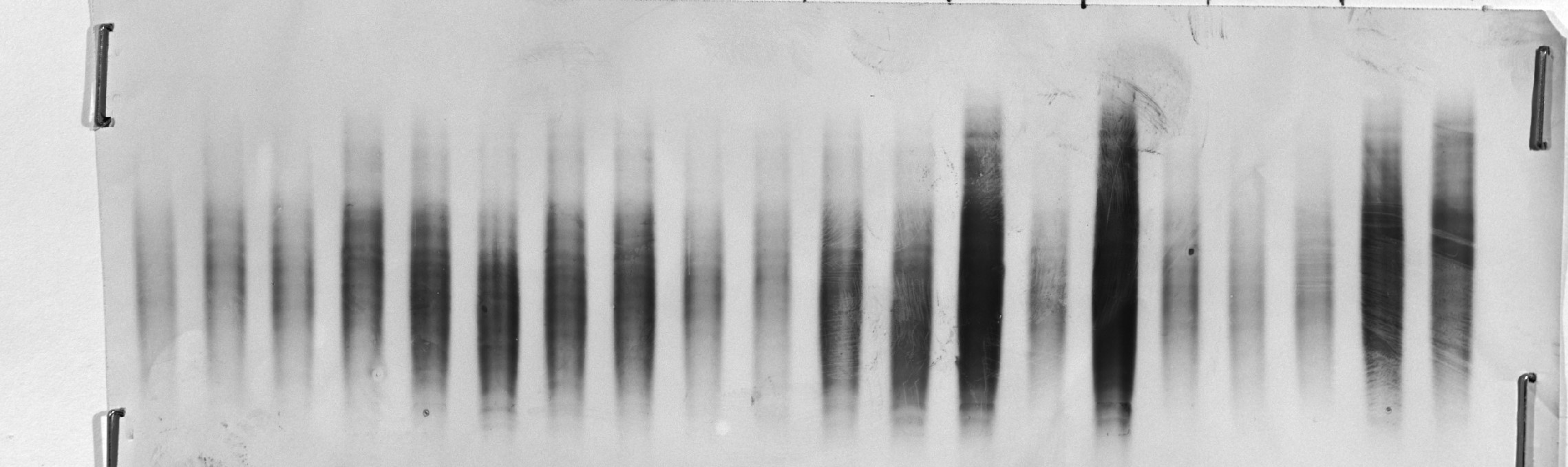


**III**

B


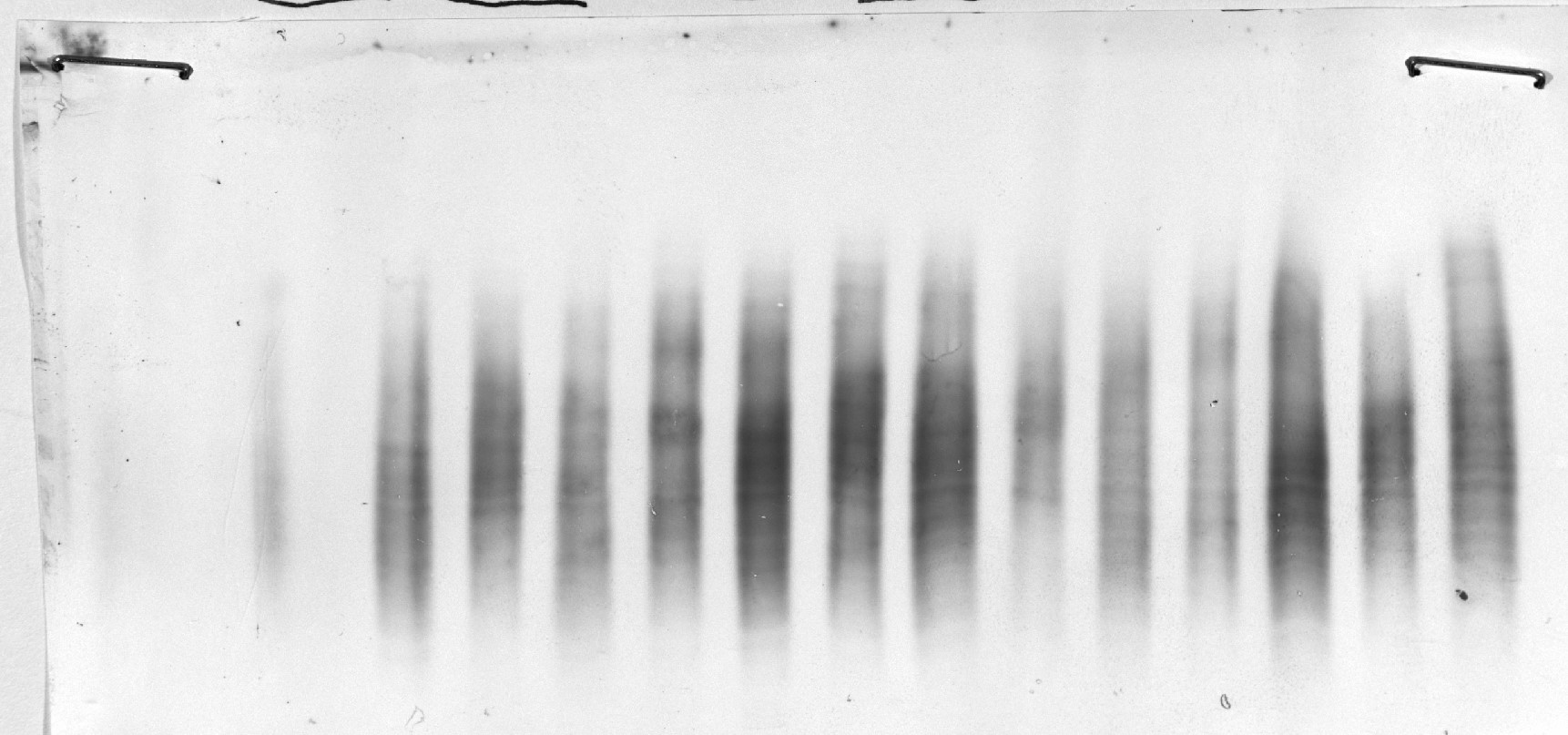


**IV**

C

**I**
