# Supplementary material for: High prevalence of intrathecal IgA synthesis in multiple sclerosis patients
Source: Sci Rep. 2022 Mar 11;12:4247. doi: 10.1038/s41598-022-08099-y (PMC8917141; doi:10.1038/s41598-022-08099-y)
Supplement: Supplementary file 2 — Supplementary Information 2. [file 41598_2022_8099_MOESM2_ESM.doc]

Supplementary Data 1.

1. Validation of the ELISA to analyze the IgA concentration in CSF and serum samples.

In order to analyze the IgA concentration in CSF and serum samples we developed a new ELISA in our laboratory. First, we validated the ELISA in order to assess the quality of the assay. We observed a high correlation between the IgA concentration and the absorbance (r= 0.940, p< 0.0001) when the concentration of this immunoglobulin ranged from 10 ng/mL to 0.3125 ng/mL (Fig. S2.1A). The multicollinearity test (VIF= 7.92; p< 0.0001) also demonstrated the linearity of the assay, and the analysis of the distribution of the residuals demonstrated the normality and homoscedasticity of the data (Fig. S1.1B-C).

Then, we calculated the coefficient of variation of the ELISA, 10.26%. In addition, the lower IgA concentration within the linear range was higher than the limit of blank (0.318 ng/mL) and the limit of detection (0.500 ng/mL).

LEGENDS:

Supplementary Figure 1. Analysis of the linearity of the quantitative ELISA. A) Study of the linear correlation of absorbances and IgA concentration. The empty circles and the whiskers represent the median and the standard deviation of the absorbances respectively and the straight line is the reference line. B) The normal P-P plot of distribution of regression standardized residual showed dots following the normality line. C) Scatterplot of the residuals showed a homogeneous distribution of the dots.

Figure S1.


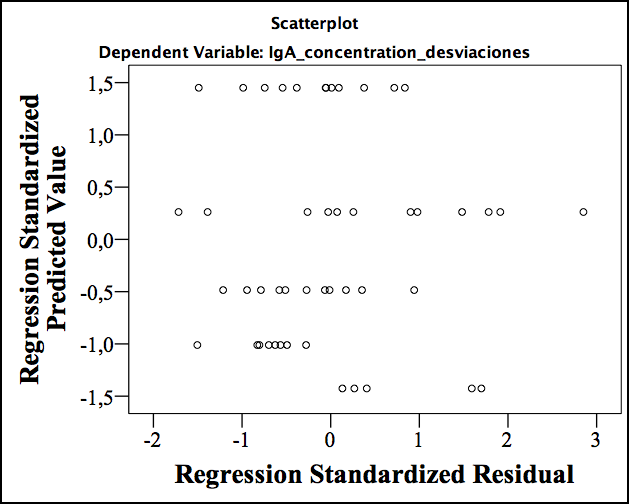

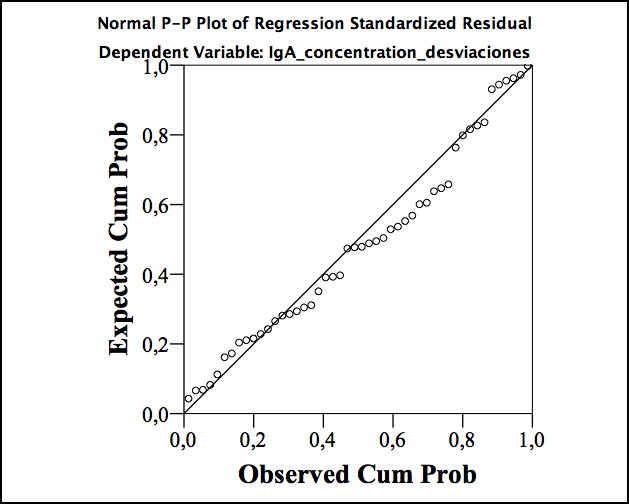


A

B

C
