# Supplementary material for: High prevalence of intrathecal IgA synthesis in multiple sclerosis patients
Source: Sci Rep. 2022 Mar 11;12:4247. doi: 10.1038/s41598-022-08099-y (PMC8917141; doi:10.1038/s41598-022-08099-y)
Supplement: Supplementary file 3 — Supplementary Information 3. [file 41598_2022_8099_MOESM3_ESM.doc]

Supplementary Data 3.

1. Validation of the isoelectrofocusing to analyze the presence of OGIgAB in CSF and serum samples.

Once achieved the best conditions to detect OGIgAB, we validated the IEF and immunodetection assay in order to assess its quality. First, we quantify the total optical density of the different dilutions of IgA applied on the gel (Fig.1) to assess the linearity of the assay, and we could observe a high correlation (r= 0.972, p< 0.0001) between the concentration and the optic density obtained (Fig S2.1A). The multicollinearity test (VIF= 12.5; p= 0.001) also demonstrated the linearity of the assay, and the analysis of the distribution of the residuals demonstrated the normality and homoscedasticity of the data (Fig. S2.1B-C). Then, we analyzed the coefficient of variation (12.83%) of the IEF and immunodetection assay.

LEGENDS:

Supplementary Figure 3. Analysis of the linearity of the IEF and immunodetection assay. A) Relation between the concentration of IgA and the optic density (O.D). The dots represent the median of the lectures and the bars the standard deviation. The line represents the reference line. B) The normal P-P plot of distribution of regression standardized residual showed dots following the normality line. C) Scatterplot of the residuals showed a homogeneous distribution of the dots.


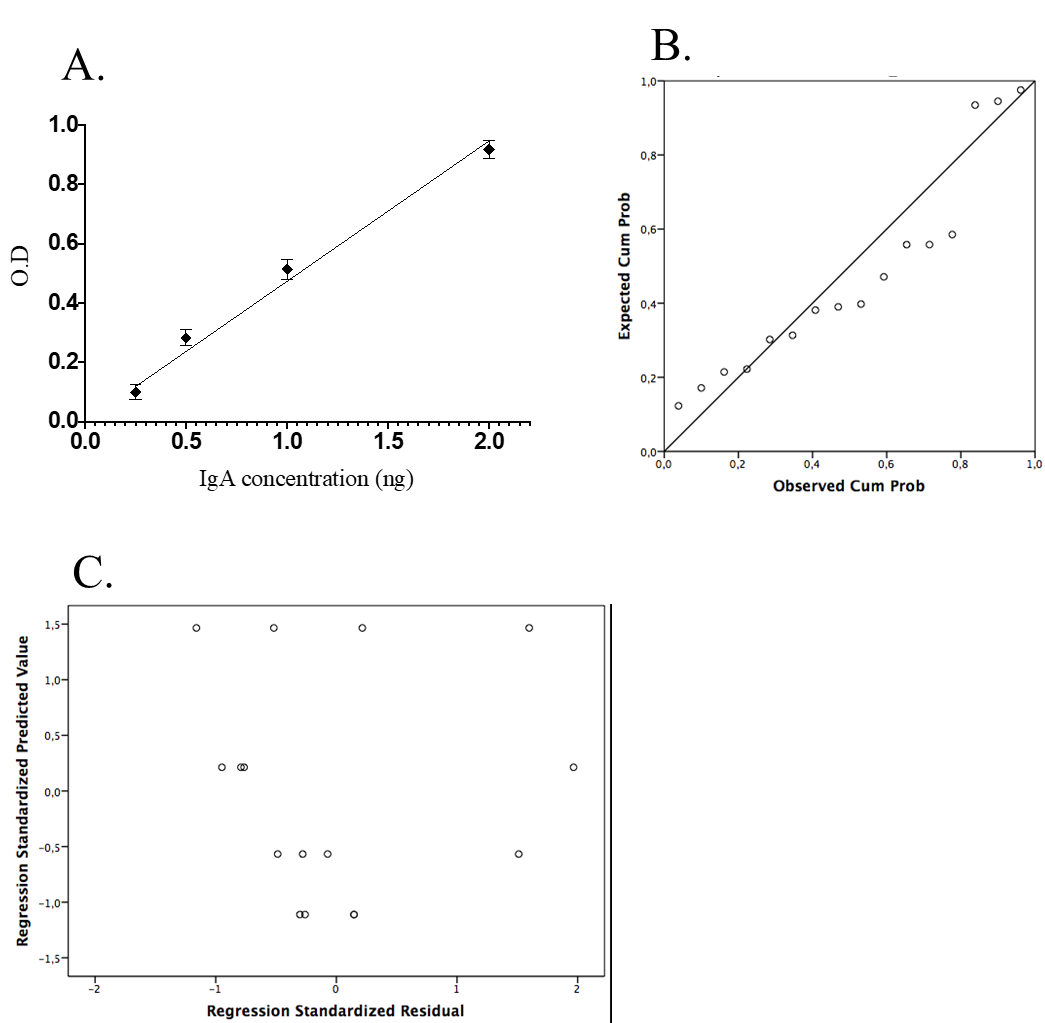
Figure S3.
